# Trifluoromethyl Thianthrenium Salts: From Organic Synthesis to Materials

**DOI:** 10.1021/acs.joc.5c02822

**Published:** 2026-01-02

**Authors:** Yingmin Ji, Roser Pleixats, Carolina Gimbert-Suriñach, Adelina Vallribera, Albert Granados

**Affiliations:** Department of Chemistry and Centro de Innovación en Química Avanzada (ORFEO-CINQA), 16719 Universitat Autònoma de Barcelona, Cerdanyola del Vallès, 08193 Barcelona, Spain

## Abstract

Trifluoromethyl thianthrenium salts have recently emerged as powerful and versatile reagents in organic chemistry, enabling the direct transfer of the CF_3_ group under mild and operationally simple conditions. Its straightforward, scalable synthesis from inexpensive starting materials provides a practical alternative to classical Umemoto- and Togni-type reagents. Beyond its role as a trifluoromethylating agent, trifluoromethyl thianthrenium salts have unlocked new classes of reagents and innovative activation modes, ranging radical, photochemical, electrochemical, mechanochemical, and even magnetoredox processes. This unique versatility has expanded the synthetic toolbox for constructing fluorine-containing molecules of interest to pharmaceuticals, agrochemicals, and functional materials. Furthermore, recent breakthroughs demonstrate that trifluoromethyl thianthrenium salts extend beyond molecular synthesis into materials settings, enabling controlled functionalization and defect passivation of two-dimensional (2D) semiconductors, thereby bridging fundamental chemical reactivity with device-level applications. This Perspective highlights the synthesis, reactivity, and emerging applications of trifluoromethyl thianthrenium salts, emphasizing how these reagents exemplify the growing interface between fluorine chemistry, radical reactivity, and materials science.

## Graphical abstract

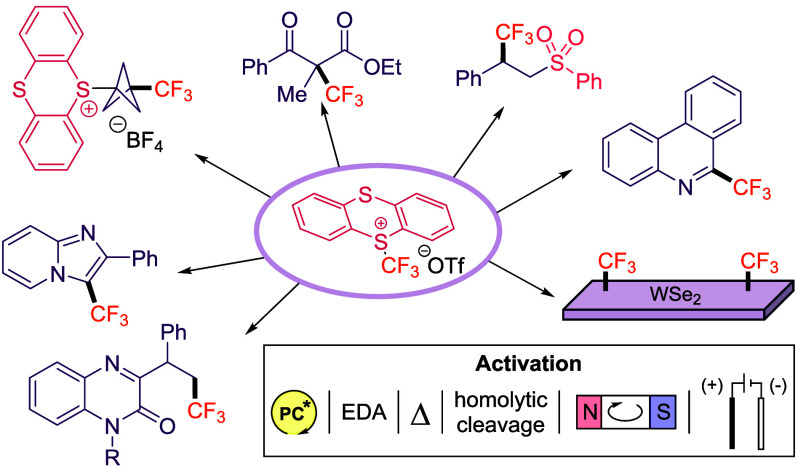

## 1 Introduction

The incorporation of the trifluoromethyl (CF_3_) group into organic molecules has become a cornerstone strategy in medicinal chemistry, agrochemicals, and materials science, owing to the unique electronic and lipophilic properties provided by this moiety. These properties have made this group a privileged motif in medicinal chemistry, where it is frequently introduced to enhance bioavailability, binding affinity, and pharmacokinetic profiles of drug candidates.^1^ It is not just a perfluoro substituted methyl but a new functional group. In agrochemical research, CF_3_-containing molecules often display improved potency and environmental persistence, while in materials science, the unique steric and electronic features of CF_3_ have been exploited to tune surface properties, optical behavior, and electronic performance.^2^ This broad relevance explains the sustained and growing interest in efficient methodologies for trifluoromethylation.

Since the first successful introduction of the trifluoromethyl group into iodobenzene derivatives in 1969 using trifluoromethyl iodide,^3^ remarkable progress has been made in the development of trifluoromethylation methodologies. Today, several distinct approaches exist for the incorporation of CF_3_ groups into organic backbones, via radical,^4^ nucleophilic,^5^ and electrophilic^6^ pathways. The expansion of this field has been enabled by the design and availability of a wide array of CF_3_-containing reagents (Figure 1),^7^ which provide access to complementary modes of reactivity.

**Figure 1:**
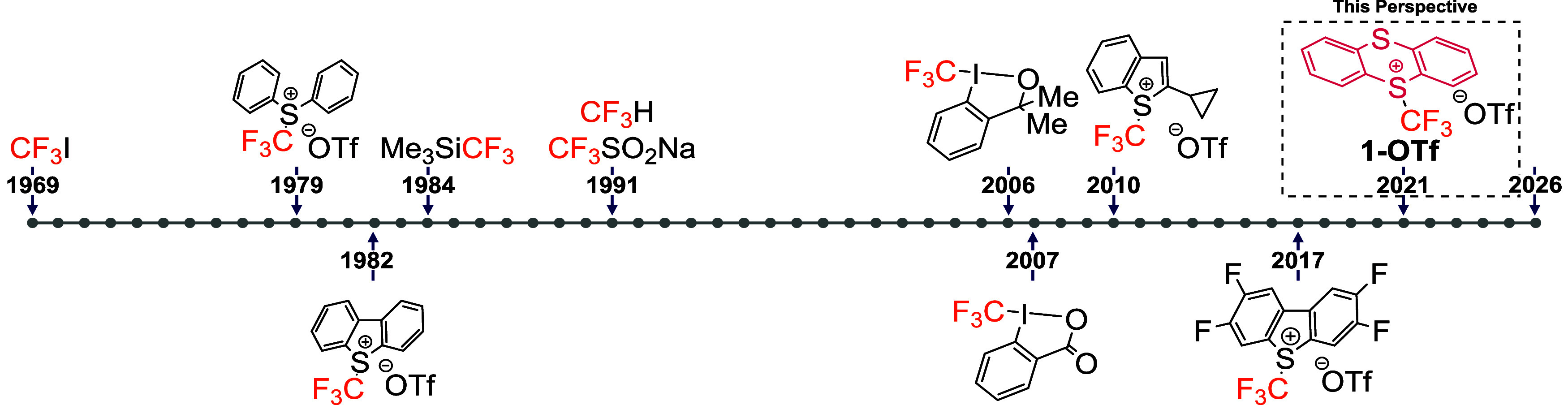
Chronological overview (1969–2026) of the development and application of trifluoromethylation reagents, highlighting selected breakthroughs across electrophilic, nucleophilic, and radical CF_3_ sources.

Among reagents used to access CF_3_^•^ species, notable examples include CF_3_I^8^ and Langlois’ reagent (CF_3_SO_2_Na)^9^ which typically under photocatalytic, thermal, or electrochemical conditions furnish CF_3_^•^. In the domain of nucleophilic reagents, silicon-based derivatives are particularly relevant, especially the Ruppert–Prakash reagent (CF_3_SiMe)^10^ as well as fluoroform (CF_3_H).^11^ Finally, electrophilic reagents encompass trifluoromethylsulfonium and trifluoromethyldiphenylsulfonium salts^12^ (early 80s’) as well as hypervalent iodine reagents^13^ such as the Togni reagents (2006–2007). These species deliver CF_3_^+^ equivalents to nucleophilic partners including enamines, enolates or heteroatom-based (O, S, N) nucleophiles. Furthermore, certain electrophilic reagents have also demonstrated their ability to generate CF_3_^•^ species via single-electron transfer (SET) pathways,^14^ thereby broadening the scope of their reactivity.

Within this context, a particularly noteworthy advance was reported by Ritter and co-workers in 2021,^15^ who developed and characterized trifluoromethyl thianthrenium triflate (TTCF_3_OTf, **1-OTf**) as a powerful and broadly applicable trifluoromethylating reagent. Remarkably, reagent **1-OTf** can be synthesized in a single step from thianthrene (TT) and triflic anhydride (Tf_2_O), providing a practical and accessible reagent. Its most distinctive feature is its multimodal reactivity: depending on the conditions, it can serve as a source of CF_3_^+^, CF_3_^•^, or CF_3_^–^, which is a rare versatility among trifluoromethylating agents. This unprecedented flexibility has enabled its application across a wide range of transformations, including electrophilic, radical, and nucleophilic trifluoromethylations. Beyond its broad substrate scope, the reagent is readily prepared in one step, stable, and easy to handle, making it a practical addition to the synthetic toolbox.

## 2 Synthesis of Trifluoromethyl Thianthrenium Salts

The synthesis of the trifluoromethyl thianthrenium triflate (**1-OTf**) is remarkably straightforward and relies on readily available and inexpensive starting materials. A one-step transformation from commercial thianthrene (TT) and the industrial feedstock triflic anhydride provides **1-OTf** in high efficiency (Scheme 1). This transformation exploits the intrinsic stability of the thianthrene radical cation (TT^•+^) and the relatively low bond dissociation energy of the newly formed S–S bond, generated upon nucleophilic attack of TT on triflic anhydride. The process is accompanied by the release of sulfur dioxide, TT^•+^ and trifluoromethyl radical, the latter two species subsequently collapse to afford **1-OTf**. Importantly, this organic backbone is obtained as a bench-stable white solid, can be stored for at least one year under ambient conditions avoiding light exposure and can be prepared in multigram scale.^15^ Remarkably, **1-OTf** is commercially available.

**Scheme 1:**
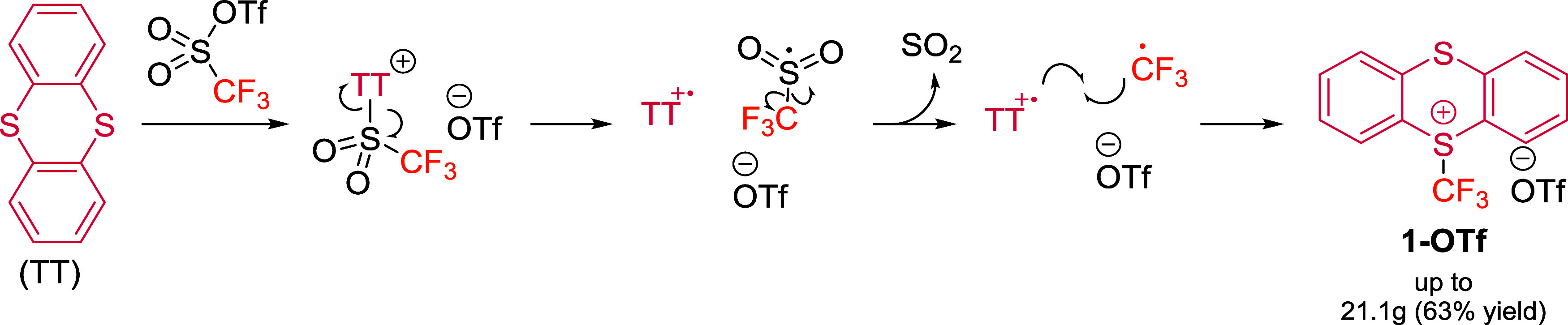
Proposed Mechanism for the Synthesis of **1-OTf** from Thianthrene (TT) and Triflic Anhydride

When compared with classical electrophilic trifluoromethylation reagents, the synthesis of **1-OTf** is remarkably efficient. For instance, Umemoto’s reagent requires a nine-step sequence to be accessed, highlighting the synthetic economy. In contrast to λ^3^-iodane-based Togni reagents, **1-OTf** displays a higher reduction potential, consistent with its cationic nature, making it a stronger outer-sphere oxidant. Furthermore, the modularity of this system is underscored by the possibility of accessing diverse thianthrenium salts (**1**) via simple anion exchange (**1-BF**_**4**_, **1-PF**_**6**_ or **1-SbCl**_**6**_). However, it is worth noting that direct exchange of TfO^–^ to NfO^–^ (NfO = C_4_F_9_SO_3_) is not efficient, reflecting the limitations of this otherwise versatile platform.

## 3 Applications in Organic Synthesis

With a robust and scalable synthesis in hand, its true potential unfolds in the realm of applications. In seminal work^15^ its versatility is demonstrated different reactivity modes (radical, electrophilic or nucleophilic) by a Cu-mediated cross-coupling with ArB­(OH)_2_, nucleophilic addition to carbonyls, Minisci-type trifluoromethylation, trifluoromethylation of thiols, hydrotrifluoromethylation of alkenes or trifluoromethylation of 1,3-diketone (Scheme 2). However, far beyond being a simple CF_3_ source, **1-OTf** has emerged as a platform reagent: a stable, scalable entry point to entirely new classes of fluorinated building blocks and as a powerful participant in photochemical transformations. The bench stability combined with radical versatility has positioned it at the crossroads of reagent design and synthetic methods.^16^

**Scheme 2:**
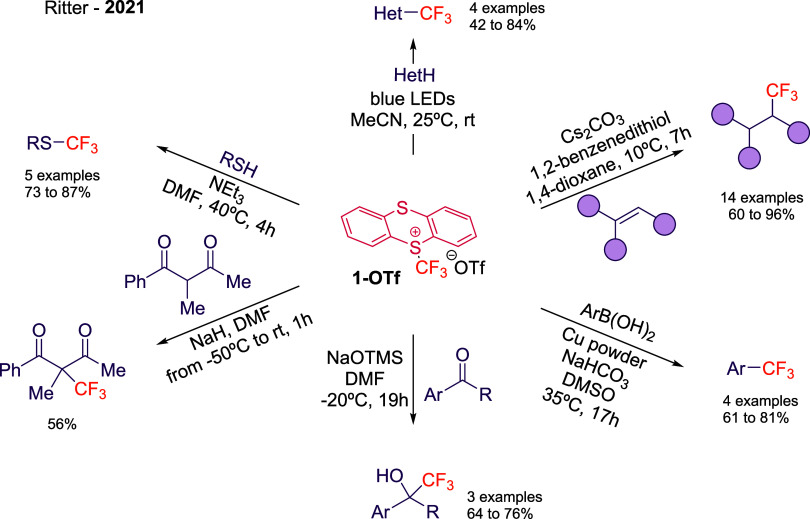
Participation of Reagent **1-OTf** in Electrophilic, Radical, and Nucleophilic Reactions

### 3.1 Precursor for New Reagents

Bicyclo­[1.1.1]­pentanes (BCPs) have emerged as valuable three-dimensional bioisosteres of phenyl rings.^17^ The replacement of 1,4-disubstituted phenyls by 1,3-disubstituted BCPs has been shown to improve the physicochemical and pharmacokinetic profiles of drug candidates. The main entrance to 1,3-disubstituted BCPs is the functionalization of the strained hydrocarbon [1.1.1]­propellane via anionic or radical addition.^18^ However, due to the low stability of this hydrocarbon, even at low temperatures, various research groups have developed strategies to construct bench-stable, yet chemically active BCP units. These include BCP derivatives such as trifluoroborates,^19^ redox-active esters,^20^ and iodides.^21^ Building on the unique reactivity of thianthrenium salts, Ritter and co-workers devised an elegant strategy in which TT-substituted BCPs serve as versatile alkylating agents.^22^ These reagents participate in radical transformations under metal and photoredox catalysis, thereby expanding the synthetic utility of BCP scaffolds. Notably, the TT-BCP-CF_3_ salt (**2**) was synthesized directly from reagent **1-OTf** and [1.1.1]­propellane under 390 nm light irradiation in acetonitrile (Scheme 3). The resulting compound can be conveniently stored under ambient conditions, representing an appealing example of a stable and bench-accessible BCP reagent.

**Scheme 3:**
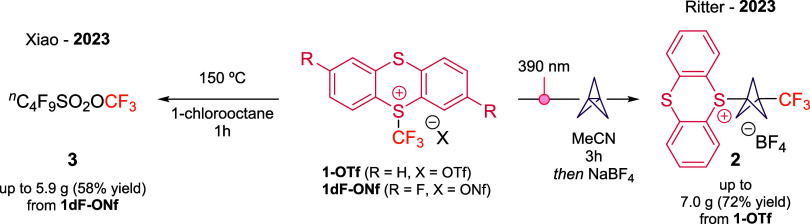
Synthesis of Reagents **2** and **3** from Trifluoromethyl Thianthrenium Salts

Moreover, the trifluoromethoxy (OCF_3_) group is a highly prized motif in medicinal chemistry, as exemplified by its presence in drugs such as Delamanid, Riluzole, Sonidegib, and Pretomanid.^23^ Its unique combination of moderate electronegativity and pronounced lipophilicity underpins its value and has accelerated the development of diverse trifluoromethoxylation reagents. Among them, RSO_3_CF_3_ (R = Ar or CF_3_) derivatives have gained prominence, as the CF_3_O^–^ can be accessed under nucleophilic activation (typically using fluorides). Recently, Hammond, Umemoto, and co-workers introduced C_4_F_9_SO_3_CF_3_ (**3**) a colorless liquid with a significantly higher boiling point (87–89 °C) compared to CF_3_SO_3_CF_3_ (19 °C).

In 2023, Lin, Jin, Xiao, and co-workers reported a practical route that utilizes **1-NfO** as precursor of the useful reagent **3** (Scheme 3).^24^ Their strategy involves sequential anion exchange from OTf^–^ to BF_4_^–^ and then to NfO^–^ to yield TTCF_3_ONf species, followed by thermolysis to release **3**, which was readily distilled and isolated on gram scale (up to 5.9 g). Notably, thianthrene was recovered and recycled. Electron-deficient thianthrenium derivatives, especially difluorinated variant (**1dF-ONf**) proved particularly effective in this work.

### 3.2 Photochemical Methods

Over the past decades, the use of light in organic synthesis has grown exponentially thanks to the renaissance of photoredox catalysis,^25^ energy transfer^26^ and EDA complexes.^27^ Compared with thermal methods, photochemical strategies often provide milder conditions, improved selectivity, and unique access to open-shell intermediates. Within this context, trifluoromethyl thianthrenium salts (**1**) have proven greatly valuable, as they combine visible light absorption with facile cleavage to generate the synthetically useful CF_3_^•^ species. The generation and application of this radical species will be discussed via homolytic cleavage, photoredox methods and EDA-complexes.

#### 3.2.1 Homolytic Cleavage of the S^+^–CF_3_ Bond

A defining property of **1-OTf** and its derivatives is their ability to absorb visible light and undergo homolytic C–S bond cleavage, delivering CF_3_^•^ together with the thianthrene radical cation (TT^•+^). This intrinsic photoreactivity has unlocked a broad range of synthetic applications (Scheme 4), where the CF_3_^•^ radical engages with diverse substrates in downstream transformations to afford high-value products.

**Scheme 4:**
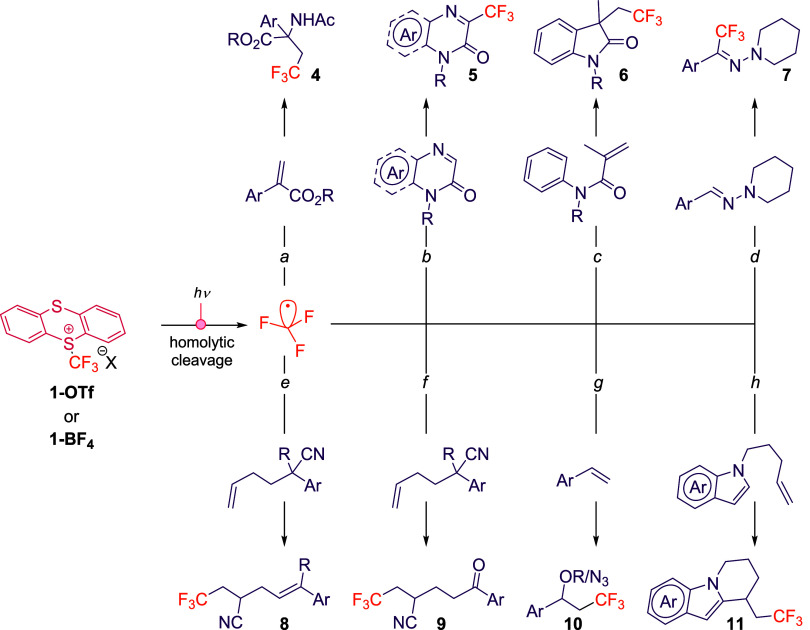
Generation of the CF_3_^•^ from **1-OTf** or **1-BF**_**4**_ under Visible-Light Irradiation via Homolytic C–S Bond Cleavage^a^

a Representative organic products obtained under the reported conditions (a–h) are shown. (a) 1 equiv of Michael acceptor, 1.5 equiv of **1-BF**_**4**_, MeCN (0.025 or 0.05 M), under purple light for 90 s. (b) 1.5 equiv of quinoxaline derivative, 1 equiv of **1-OTf**, 1 equiv of NaH_2_PO_4_, acetone (0.1 M), under 395 nm light for 12 h. (c) 1.5 equiv of acrylamide, 1 equiv of **1-OTf**, 1 equiv of K_2_CO_3_, MeCN (0.1 M), under 395 nm light for 12 h. (d) 1.5 equiv of hydrazone derivative, 1 equiv of **1-OTf**, 1 equiv of K_2_CO_3_, MeCN (0.1 M), under 395 nm light for 12 h. (e) 1 equiv of radical acceptor, 1.5 equiv of **1-OTf**, 0.1 equiv of Cu­(MeCN)_4_PF_6_, MeCN (0.1 M), under blue light for 12 h. (f) 1 equiv of radical acceptor, 2 equiv of **1-OTf**, 3 equiv of H_2_O, MeCN (0.1 M), under blue light for 24 h. (g) 1 equiv of alkene, 1.5 equiv of **1-OTf**, 5 equiv of alcohol in DCE (0.1 M) or 3.5 equiv of TMSN_3_ in DCM (0.1 M), under 390 nm light for 2 h. (h) 1 equiv of alkene, 1.5 equiv of **1-OTf**, DMSO (0.1 M), under blue light for 6 h.

A seminal contribution in this area was reported by Ritter and co-workers,^28^ who demonstrated the rapid trifluoromethylation of Michael acceptors under 390 nm irradiation using **1-BF**_**4**_. Remarkably, the transformation proceeded within 90 s to furnish α- and β-amino acid derivatives (**4**) with broad functional group tolerance, including esters, ethers, and late-stage functionalized scaffolds (Scheme 4, conditions a). Mechanistic studies supported a sequence involving CF_3_^•^ addition to alkene, radical recombination with TT^•+^, and subsequent nucleophilic capture.

Building on this foundation, Xiang, Zhang, and Wang^29^ extended the scope of phototriggered homolysis to a wider array of substrates, including (hetero)­arenes (**5**, Scheme 4, conditions b), acrylamides (**6**, Scheme 4, conditions c) and hydrazones (**7**, Scheme 4, conditions d). Their work combined experimental and computational evidence to confirm that direct C–S bond cleavage, rather than EDA complex formation, governs the initiation step. This clarified the mechanistic landscape and broadened synthetic access to trifluoromethylated products under mild conditions.

Expanding the reactivity space, Huang and colleagues^30^ introduced a cascade process wherein CF_3_^•^ addition to terminal alkenes triggers a cyano migration using catalytic Cu­(MeCN)_4_PF_6_. The outcome can be tuned to afford either alkenyl nitriles (**8**, Scheme 4, conditions e) or ketones (**9**, Scheme 4, conditions f) depending on the presence or absence of water, underscoring the versatility of **1-OTf** in radical cascades.

We have recently explored this type of reactivity, developing a difunctionalization of alkenes (Scheme 4, conditions g) that installs both a trifluoromethyl group and a second nucleophilic fragment (alkoxy, hydroxy, or azide, **10**).^31^ This mild and rapid protocol tolerates a wide substrate scope—including substituted alkenes, alcohols, esters, ethers, and heteroarenes—and highlights the potential of **1-OTf** to orchestrate multicomponent radical processes.

In a complementary vein, Wu and co-workers^32^ leveraged the same radical generation strategy in a trifluoromethylation/cyclization sequence, enabling the rapid construction of tricyclic indoles from unactivated alkenes via CF_3_^•^ addition, intramolecular cyclization, and SET-driven carbocation formation from TT^•+^ (**11**, Scheme 4, conditions h).

Further diversification was showcased by Guo and Xia,^33^ who reported the vinylation of propargyl alcohols to deliver trifluoromethylated alkenes through a radical cascade involving hydrogen atom transfer and cyclization (**12–14** in Scheme 5). Under purple light, C–S bond homolysis generates CF_3_^•^ and TT^•+^ radical species. The CF_3_^•^ triggers a cascade addition, 1,5-HAT, functional group migration (FGM), followed by SET and deprotonation, ultimately affording the desired product (Scheme 5).

**Scheme 5:**
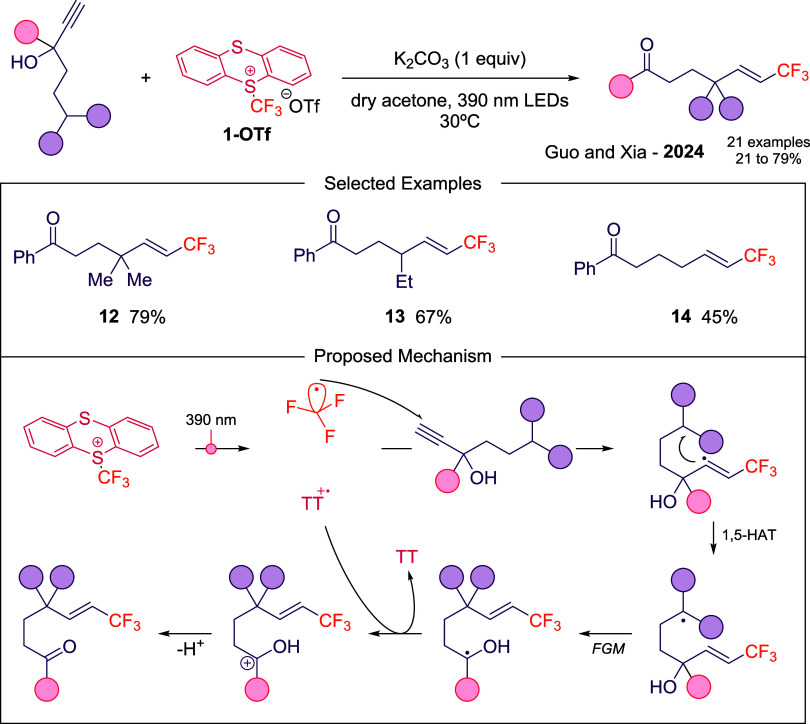
Photoinduced Regioselective Vinylation of Csp^3^–H Bonds Using **1-OTf** Described by Guo and Xia

Collectively, these studies demonstrate how the photochemical homolysis of **1-OTf** has evolved from proof-of-concept radical generation into a general strategy for designing trifluoromethylation and cascade processes. The balance of stability, light responsiveness to purple and blue LEDs, and radical reactivity makes **1-OTf** a unique entry point for the development of new transformations in fluorine chemistry.

#### 3.2.2 Formation of EDA Complex

Another metal-free strategy for trifluoromethyl radical generation from **1-OTf** relies on the formation of electron donor–acceptor (EDA) complexes. The intrinsic cationic character of thianthrenium salts makes them excellent electron acceptors,^34^ capable of pairing with suitable donors to form light-absorbing aggregates. Upon visible-light excitation, charge transfer within the complex can induce mesolytic cleavage of the C–S bond, releasing CF_3_^•^ and neutral TT (Scheme 6, top).

**Scheme 6:**
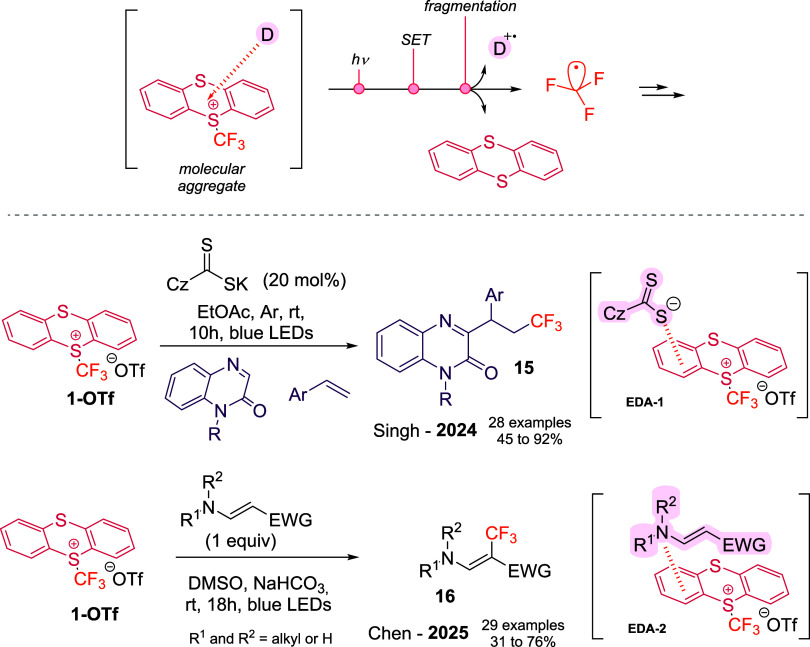
Generation of CF_3_^•^ from **1-OTf** via Charge-Complex Photoirradiation with Suitable Electron-Donors (**D**)­^a^

a Representative organic products obtained under the reported conditions by Singh and Chen. Cz = carbazole. EWG = electron-withdrawing group.

In 2024, Singh and co-workers^35^ reported the first trifluoromethylation via EDA complex formation, developing a three-component difunctionalization of alkenes. A carbazolyl dithiocarbamate served as catalytic electron-donor, forming an EDA complex with **1-OTf** (**EDA-1** in Scheme 6). Upon irradiation, cleavage produced TT and a CF_3_^•^, which underwent Giese-type addition to the unsaturated substrate. The resulting radical intermediate was subsequently trapped by quinoxalinone, followed by 1,2-hydrogen migration and deprotonation, delivering the final product **15**.

In a complementary direction, Chen and co-workers^36^ showed that amines can act as donors enabling trifluoromethylation of primary and secondary amines, proceeding through **EDA-2** in Scheme 6. Visible-light excitation triggered mesolytic cleavage, affording enamines through a radical recombination–deprotonation pathway (**16**, Scheme 6, bottom).

Both studies support the formation of a new molecular aggregate through the red-shift observed in the UV/vis experiment and illustrate the potential of EDA complexes as catalyst-free photochemical manifolds for trifluoromethylation, providing additional reactivity modes to those accessible through direct homolysis.

#### 3.2.3 Photoredox and Metallaphotoredox Methods

Photoredox catalysis offers another powerful strategy for unlocking the reactivity of **1-OTf**. In this approach, both transition-metal complexes or organic dyes photocatalysts (PC) can undergo SET with the thianthrenium reagent, producing CF_3_^•^ under mild conditions. A key advantage is the modularity of the process: depending on reaction conditions, both oxidative and reductive quenching pathways can be engaged (Scheme 7), and dual catalysis with metals can unlock further transformations.

**Scheme 7:**
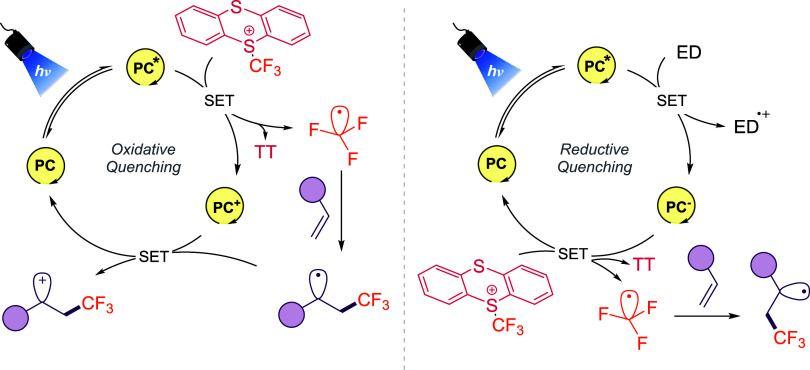
Oxidative and Reductive Quenching Pathways in Photoredox Catalysis with **1-OTf** ^a^

a ED = electron-donor. SET = Single-electron transfer. TT = thianthrene. PC = photocatalyst.

The first demonstration came from Chen and Wu,^37^ who employed an iridium photocatalyst in combination with **1-OTf**, alkynes, SO_2_, and hydrazines to access vinylsulfonohydrazides. The mechanism involves SET from the excited PC to **1-OTf**, generating CF_3_^•^, which adds to the alkyne. Subsequent capture by SO_2_ and radical–radical coupling with a hydrazine-derived radical furnishes **17**, while PC turnover is achieved via hydrazine oxidation (Scheme 8).

**Scheme 8:**
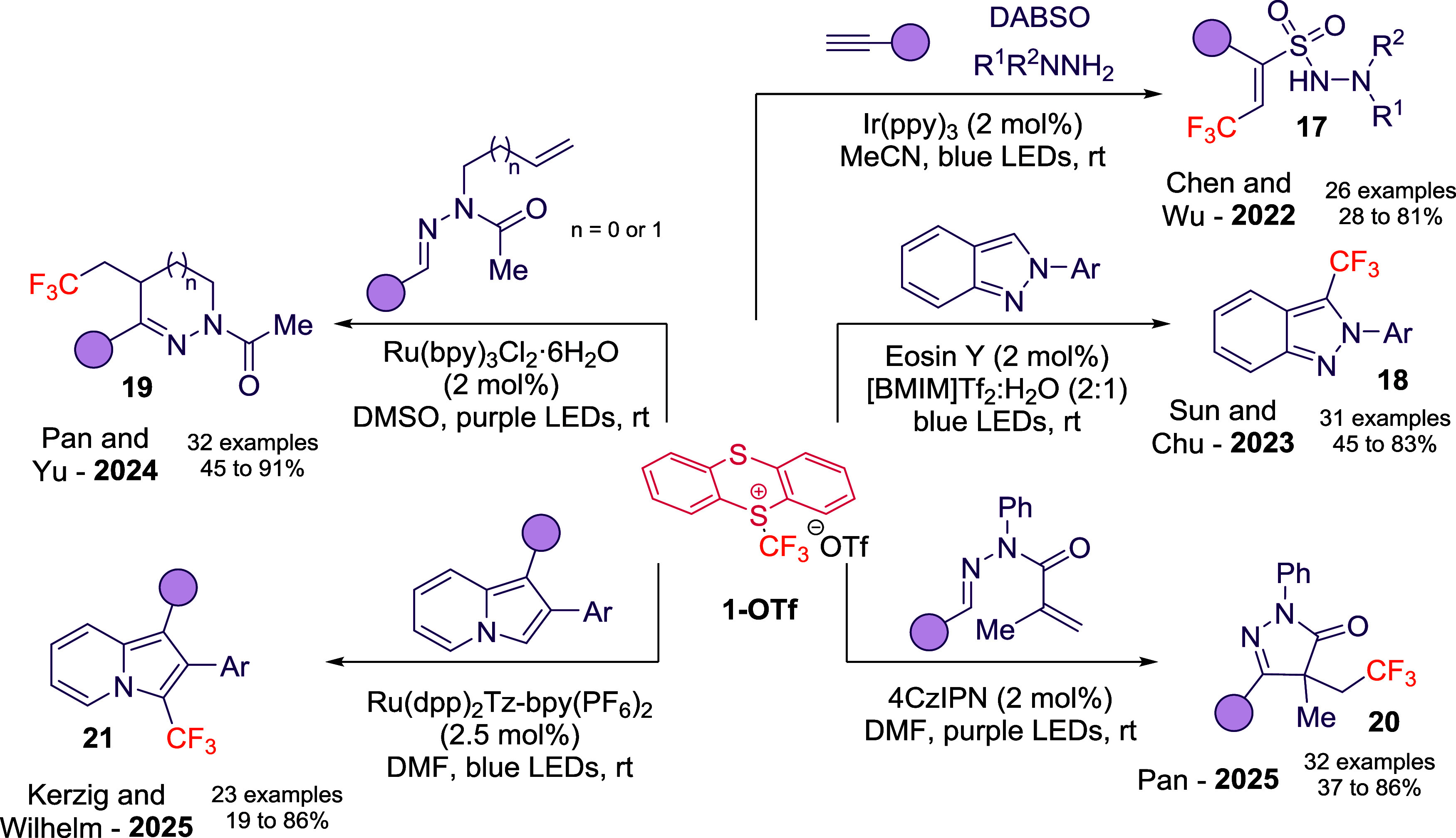
Photoredox Reactivity of **1-OTf** ^a^

a bpy = 2,2′-bypyridine. dpp = tris­(4,7-diphenyl-1,10-phenanthroline). ppy = 2-phenylpyridine. DABSO = 1,4-diazabicyclo[2.2.2]­octane bis­(sulfur dioxide) adduct. [BMIM]­NTf_2_ = 1-hexyl-3-methylimidazolium bis­(trifluoromethanesulfonyl)­imide.

In 2023, Sun and Chu described the trifluoromethylation of 2H-indazoles using **1-OTf** under Eosin Y photocatalysis.^38^ A key feature of the method is the use of [BMIM]­NTf_2_/H_2_O ionic-liquid mixtures as recyclable reaction media, enabling up to five reuse cycles with minimal loss in yield. The transformation displays broad functional-group tolerance and provides access to bioactive derivatives. Mechanistic studies support a radical pathway initiated by SET from excited Eosin Y to **1-OTf** to yield compounds **18** (Scheme 8).

Shortly after, Pan and Yu developed a ruthenium-catalyzed protocol enabling the synthesis of CF_3_-containing hydrazolones and hydropyridazines (**19**, Scheme 8) from *N*-allyl and *N*-homoallyl aldehyde hydrazones. Supported by Stern–Volmer experiments, their mechanistic model involves CF_3_^•^ generation via SET, Giese-type addition, and intramolecular cyclization, followed by oxidation of the resulting *N*-centered radical by PC^+^.^39^ Control experiments avoiding the use of Ru under the optimal reaction conditions are omitted and an homolytic cleavage may not be discarded.

Expanding beyond transition-metal catalysts, Pan and co-workers^40^ later employed 4CzIPN (1,2,3,5-tetrakis­(carbazol-9-yl)-4,6-dicyanobenzene) photocatalyst to achieve intramolecular cyclization of *N*-methacryloyl aldehyde hydrazones, affording pyrazol-5-ones (**20**, Scheme 8). This method displayed broad functional group tolerance—including halides, ethers, and heteroarenes—and highlighted the synthetic flexibility of organic PCs in **1-OTf** chemistry. Interestingly, control experiments avoiding the use of 4CzIPN under the optimal reaction conditions are omitted and homolytic cleavage may not be discarded. Indeed, Zhao, Yang, and He reported the same transformation, achieved through direct homolytic C–S bond cleavage from **1-OTf** under 415 nm irradiation in ethanolic solution.^41^

In parallel, Kerzig and Wilhelm showcased the late-stage trifluoromethylation of indolizines (**21**, Scheme 8) using bichromophoric ruthenium complexes.^42^ Their results emphasized how careful photocatalyst design can deliver high chemoselectivity, scalability, and compatibility with complex heteroaromatic frameworks.

A particularly striking advance was reported by Das and co-workers, who demonstrated wavelength-controlled regioselectivity in the radical difunctionalization of alkenes using **1-OTf** and sulfinates.^43^ Under red-light-mediated copper/photoredox dual catalysis with Os-based PC, the reaction outcome was inverted: instead of classical β-trifluoromethylation, α-trifluoromethylation was achieved, coupled with β-sulfonylation (**22–25**, Scheme 9). Mechanistic analysis revealed that long-wavelength irradiation selectively excited the photocatalyst rather than **1-OTf**, reshaping the SET pathway and enabling this regioselectivity switch. Conceptually, this study illustrates how photonic energy tuning can open new reactivity landscapes, offering a powerful paradigm for designing otherwise inaccessible trifluoromethylation patterns.

**Scheme 9:**
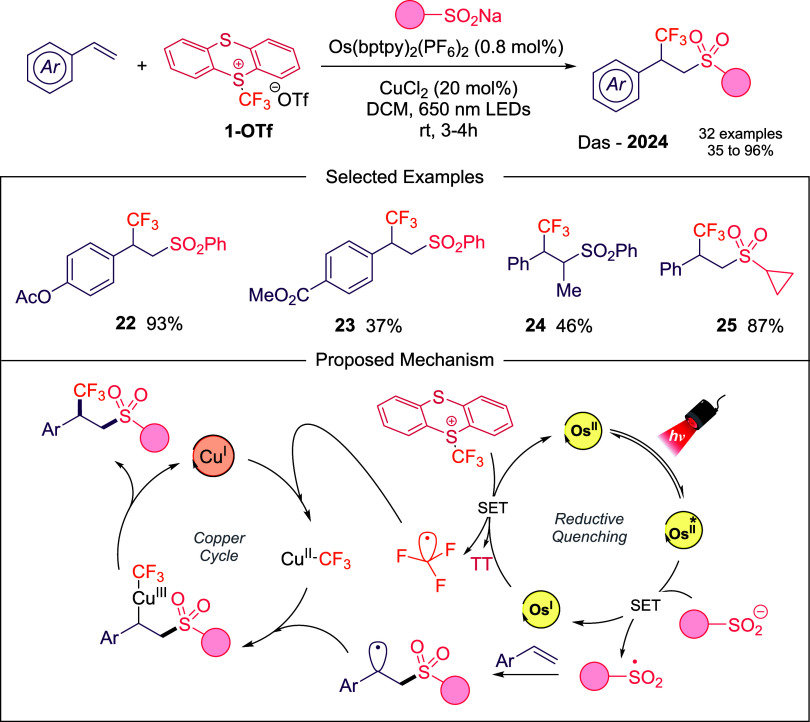
Photochemical Sulfonyltrifluoromethylation of Olefins Described by Das’s Group

A recent contribution by Huo and co-workers describes a photoredox net-neutral three-component radical cascade to access elaborated glycine derivatives (Scheme 10).^44^ The reaction proceeds using Eosin Y as photocatalyst and notably, the reaction can also proceed in the absence of a photocatalyst albeit with lower efficiency, consistent with the formation of an EDA complex between the glycine starting material and **1-OTf**. The transformation is able to accommodate diverse glycine derivatives, from activated to unactivated alkenes (**26**–**28**). The mechanism is supported by radical trapping, fluorescence quenching studies, light on–off experiments and photochemical quantum yield.

**Scheme 10:**
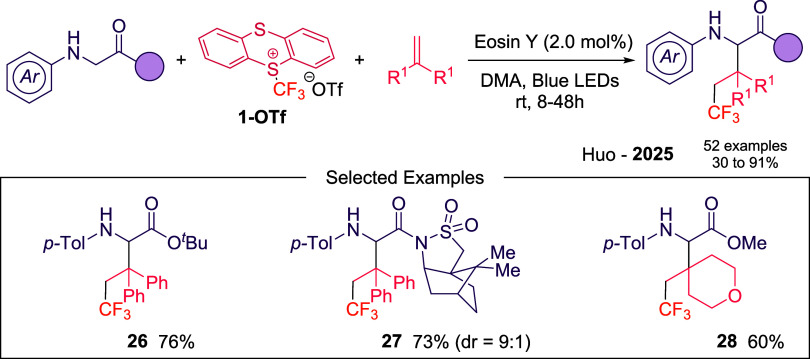
Alkyl-Trifluoromethylation of Glycine Derivatives Described by Huo’s Laboratory

### 3.3 Electrochemical Approaches

Electrochemistry has emerged as a powerful and versatile tool in organic synthesis, attracting increasing attention due to its numerous advantages, including mild reaction conditions, facile tunability of redox potentials, and scalability. Moreover, the use of electricity as a green and sustainable alternative to traditional redox reagents—often costly and hazardous—represents a significant step forward in promoting environmentally responsible approaches to synthesis and catalysis.^45^ In this regard, trifluoromethyl thianthrenium salts have also found a valuable role within electrochemical activation platforms.

Last year, the difunctionalization of alkenes and alkynes using quinoxalinones and trifluoromethyl thianthrenium triflate was reported using electrochemistry.^46^ The work presented by Zeng and colleagues represents the first example using electrochemical conditions to activate trifluoromethyl thianthrenium reagents. The reaction proceeds efficiently in an undivided cell equipped with carbon felt as both the anode and cathode, operating at a constant current of 5 mA and a temperature of 70 °C. This methodology enables access to a diverse array of trifluoromethylated alkylquinoxalinones (**29**, Scheme 11), demonstrating broad functional group tolerance. Mechanistic studies suggest that **1-OTf** serves as a bifunctional reagent, acting both as a source of CF_3_^•^ species and as an electrophilic mediator (via the thianthrene moiety), facilitating a key oxidative step in the reaction mechanism.

**Scheme 11:**
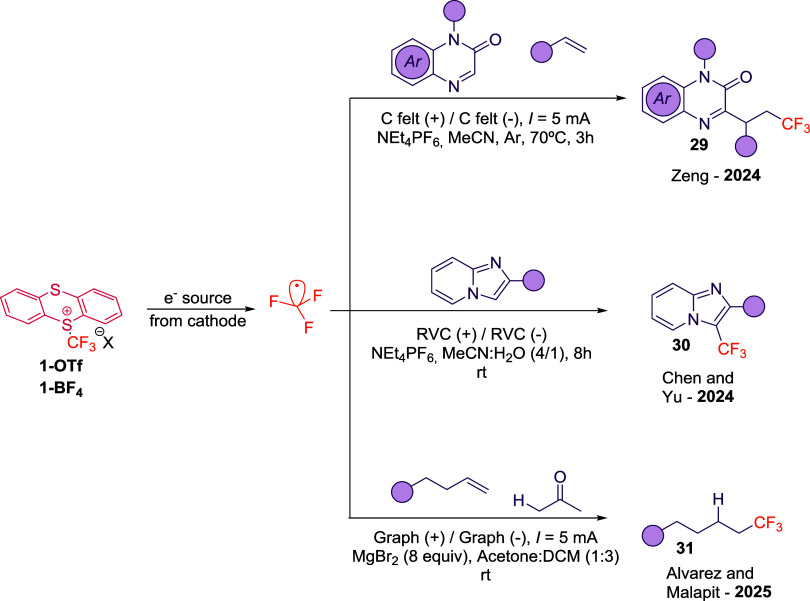
Electrocatalytic Construction of CF_3_-Containing Frameworks from Reagent **1-OTf** ^a^

a RVC= glassy carbon foam electrodes. Graph = graphite.

In the same year, Chen and Yu developed an electrochemical approach to access trifluoromethylated imidazole-fused heterocycles (**30**, Scheme 11).^47^ The reaction was carried out in an undivided cell equipped with two glassy carbon foam electrodes (RVC) under a constant current of 5 mA. This synthetic method exhibited a broad substrate scope, accommodating structurally diverse substrates, including functional groups such as nitriles, halides, sulfones, and phenols. Notably, the authors demonstrated the scalability of the protocol up to a 5 mmol scale. Mechanistic investigations, including radical trapping and control experiments, indicated that the generation of CF_3_^•^ radicals occurs at the cathode via reduction of the **1-OTf** reagent. Concurrently, the imidazole-fused substrate undergoes single-electron oxidation at the anode, forming a radical cation that subsequently engages in a radical cross-coupling with CF_3_^•^ to furnish the desired product **30** (Scheme 11).

Recently, Alvarez and Malapit reported the hydrotrifluoromethylation of unactivated olefins under electrochemical conditions (**31**, Scheme 11).^48^ A key to the success of this transformation was the use of MgBr_2_ as a sacrificial bromide source, which presents an onset oxidation potential at +0.20 V vs Fc/Fc^+^. Notably, the redox system exhibited a remarkably narrow operating window of ∼1.00 V, governed by the reduction potential of reagent **1-OTf**. This narrow potential window was crucial in minimizing undesired redox side reactions, while preserving many functional groups. In addition, during reaction optimization, a pronounced counterion effect was observed. Specifically, while the sulfonium salt bearing a BF_4_^–^ (**1-BF**_**4**_) counterion afforded the desired product, **1-OTf** did not. This outcome was attributed to differences in electrode potential. While the **1-OTf** led to elevated electrode potentials due to the low solubility of MgBr_2_, which in turn promoted undesired anodic side reactions, **1-BF**_**4**_ enhanced the solubility of the magnesium salt via strong ion pairing with Mg^2+^. This interaction reduced electrode resistance and ultimately enabled an efficient hydrotrifluoromethylation reaction. Interestingly, the ketone substrate is the hydrogen source to yield the final product.

Lastly, **1-OTf** is used as one of the radical precursors the organonickel platform for C­(sp^2^)–C­(sp^3^) bond formation described by Kalyani and Sevov.^49^ Under electroreductive conditions persistent Ni^II^–Ar complexes can be generated, and **1-OTf** behaves as an efficient electrophilic precursor to CF_3_^•^, enabling C­(sp^2^)–CF_3_ coupling through radical capture at Ni^II^–Ar intermediates (Scheme 12). Stoichiometric mechanistic studies suggests that **1-OTf** delivers the radical fragment upon single-electron reduction, and the resulting Ni^III^ species rapidly collapses to forge the C–CF_3_ bond. The method enables telescoped trifluoromethylation of aryl chlorides, heteroaryl bromides, and vinyl triflates, which typically are challenging organic skeletons to engage using catalytic cross electrophile coupling conditions due to mismatched reactivity. The mild set of electrochemical conditions tolerates sensitive functionalities such as free OH, CO_2_H, amines, heterocycles and complex drug-like scaffolds (**32**-**34**). Importantly, the authors show that trifluoromethylation proceeds smoothly in miniaturized formats.

**Scheme 12:**
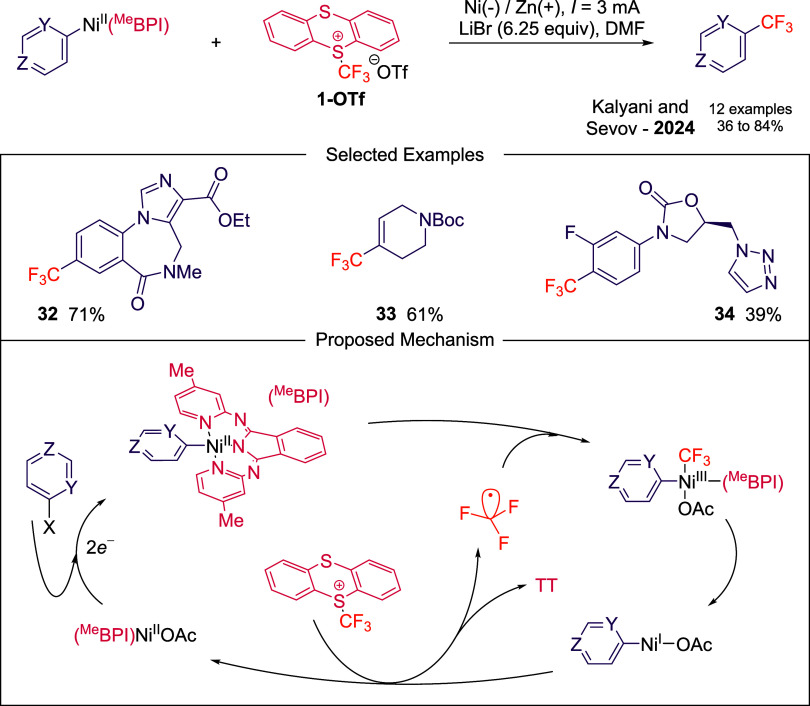
Electrochemical Trifluoromethylation of Organonickel Complexes Described by Kalyani and Sevov

### 3.4 Electromagnetic Induction

Electromagnetic induction has recently emerged as a complementary activation mode to light-driven and mechanochemical strategies for redox transformations. It is based on the use of a rotating magnetic field and steel rods, under mechanochemical impact. The concept relies on the temporal generation of highly polarized particles by piezoelectric materials which can transfer electrons via SET events.^50^ Thus, through this electromagnetic reduction, electrons will be transferred to organic molecules. Optimization of these reactions is related to magnetic intensity, rotating frequency and rod length, as well as classical solvent and reagent’s equivalent screening.

In 2024, Li and co-workers reported the first magnetoredox-mediated trifluoromethylation of heteroarenes (**35**) and hydrotrifluoromethylation of alkenes (**36**) with **1-OTf**.^51^ When heterocycles were exposed to **1-OTf** in acetonitrile under a rotating magnetic field, efficient and highly regioselective incorporation of the CF_3_ unit was achieved in electron-rich substrates such as indoles and pyrroles (Scheme 13). In contrast, electron-deficient heteroarenes such as pyridines and pyrimidines remained unreactive. The same system enabled hydrotrifluoromethylation of alkenes under aerobic conditions, with water serving as a hydrogen atom donor. Monosubstituted and 1,1-disubstituted olefins reacted smoothly, affording moderate to good yields while tolerating a variety of functional groups. Mechanistic studies supported a pathway involving reduction of **1-OTf** via electromagnetic induction (Scheme 13, top), followed by radical generation and subsequent classical CF_3_^•^ reactivity.

**Scheme 13:**
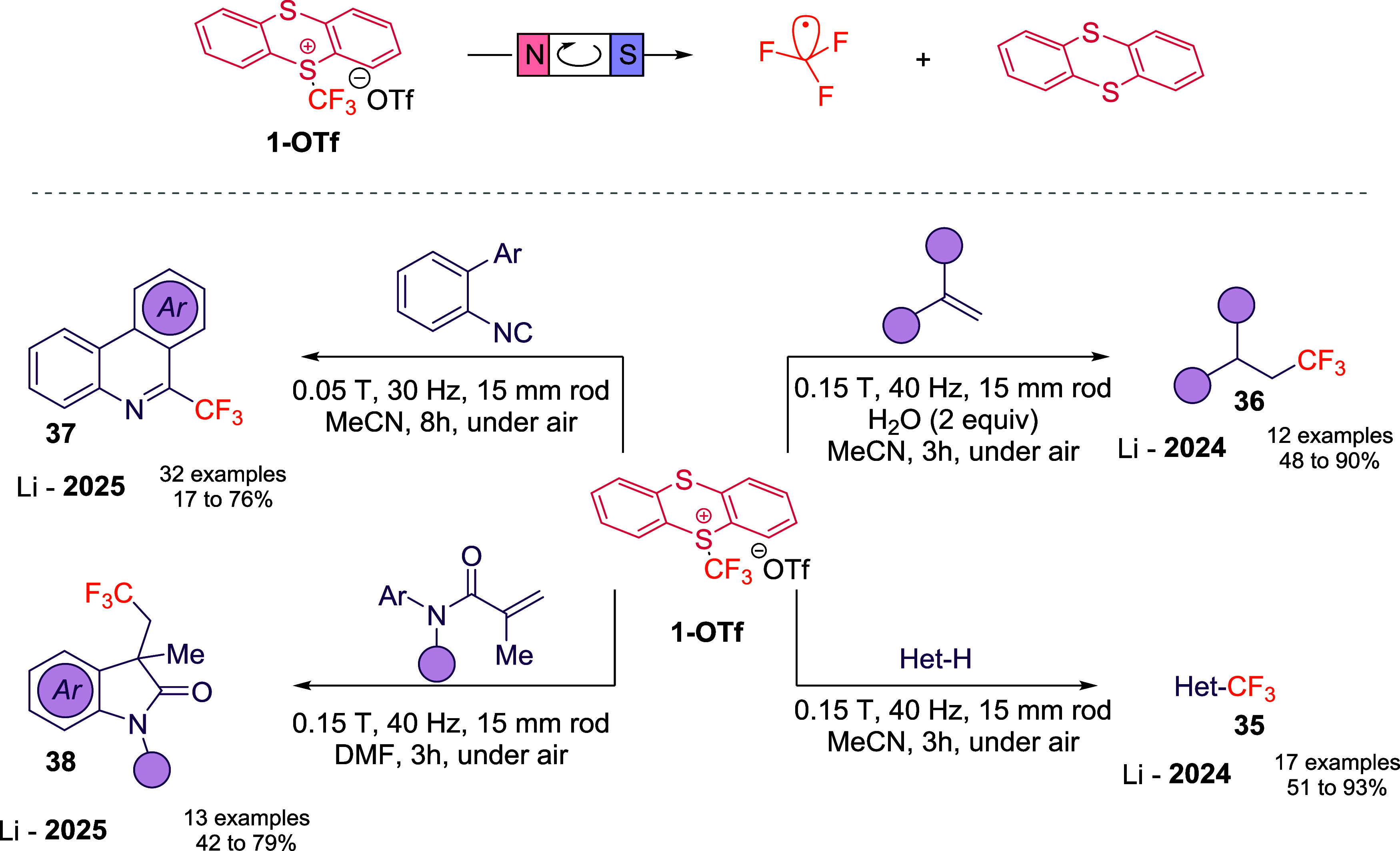
Mapping the Trifluoromethylated Chemical Space of **1-OTf** by Magnetoredox Activation

Very recently, Li’s group further expanded the scope of magnetoredox trifluoromethylation to isocyanides and *N*-arylacrylamides (Scheme 13, left).^52^ Reactions of **1-OTf** with isocyanides furnished trifluoromethylated phenanthridines (**37**) in good yields, and the method was successfully translated to gram scale, underscoring its preparative utility. Moreover, the well-established trifluoromethylation of *N*-arylacrylamides to yield trifluoromethylated oxindoles (**38**) was efficiently triggered by a rotating magnetic field, providing a mild and operationally simple alternative to photochemical initiation.

Overall, these studies highlight electromagnetic induction as a novel activation paradigm for **1-OTf**, broadening the toolbox of external energy inputs capable of mediating radical trifluoromethylation. By circumventing the need for catalysts or additives, this strategy aligns with the principles of green chemistry and opens a promising frontier for sustainable fluorine chemistry.

### 3.5 Substrate-Triggered SET Trifluoromethylation

The installation of the CF_3_ group on quaternary Csp^3^ centers is highly valued in drug design due to enhanced steric and electronic control. Our group^53^ further explored the reactivity of **1-OTf** on acyclic 3-oxoesters using ^*t*^BuOK under 35 °C (Scheme 14). Mechanistic studies indicate a radical-mediated SET pathway, in which enolate attacks **1-OTf** and forms a sulfurane intermediate, followed by CF_3_^•^ generation, which adds to the enolate and delivers final 3-oxoesters via radical chain process. Radical trapping experiments confirm the intermediacy of CF_3_^•^. This strategy exhibits broad substrate scope, tolerating alkynyl, aryl, alkyl, sterically demanding, and heteroaromatic substituents, providing a practical route to quaternary Csp^3^–CF_3_ motifs (**39**).

**Scheme 14:**
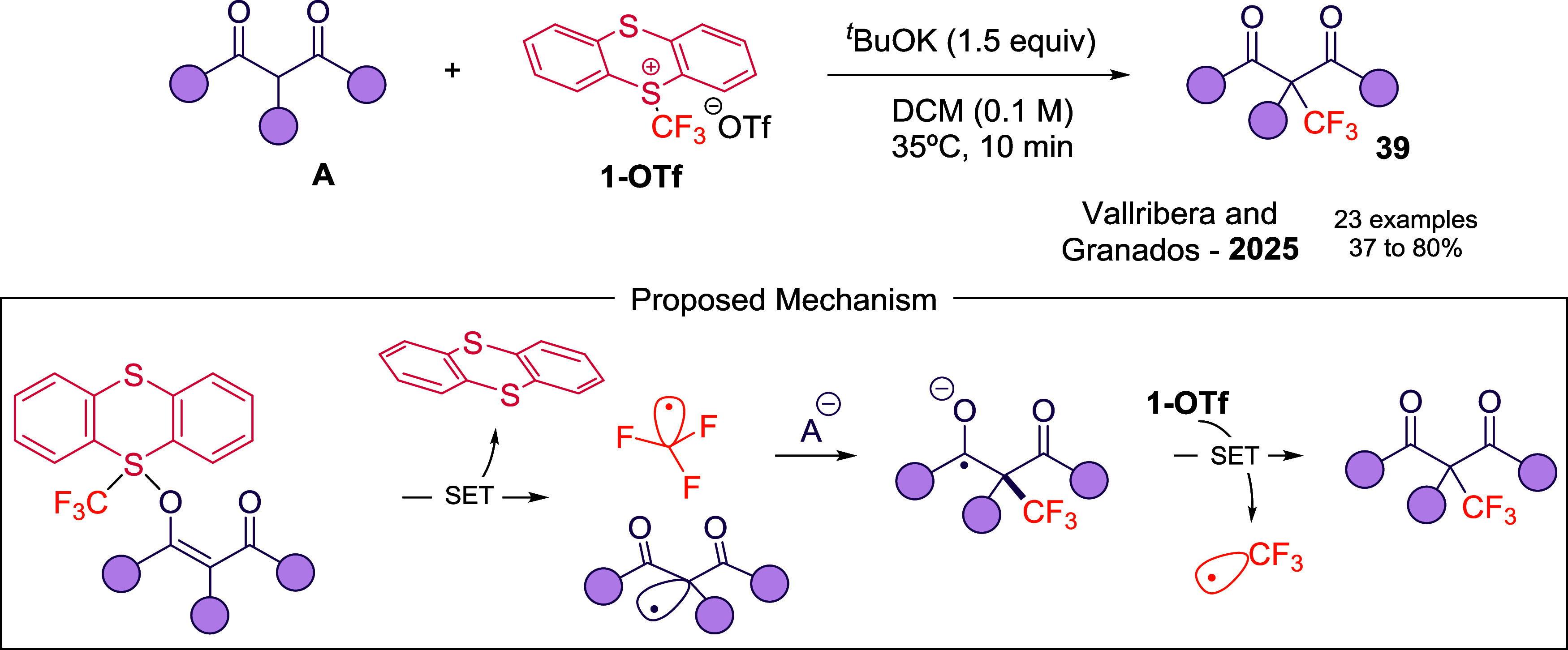
Direct Access to Quaternary CF_3_-Substituted Centers from 3-Oxocarbonyls

## 4 Applications for Materials and Polymers

The integration of 2D materials with molecular chemistry to generate molecule–2D material heterostructures presents a compelling strategy for advancing material design and applications.^54^ While physisorbed molecules can induce doping through charge transfer, covalent functionalization provides greater advantages. Many covalent strategies rely on harsh conditions that risk damaging the lattice of the material. Recent advances using mild electrophiles such as maleimides demonstrated transition metal dichalcogenides (TMDs) can be functionalized under mild conditions, motivating the research for new surface modifications.^55^ In the context of materials applications using **1-OTf**, two recent reports underscore its versatility in enhancing the electronic performance of TMDs. In 2024, trifluoromethylation was established as a mild and general functionalization pathway for WSe_2_ and MoS_2_, yielding tunable p-type doping along with modulation of optical properties.^56^ This work validated the conceptual role of trifluoromethylating agents in tailoring both interfacial and bulk properties of layered materials. Building on this foundation, in 2025 **1-OTf** was employed directly to passivate defects in WSe_2_, thereby reducing contact resistance and enabling efficient p-type charge injection in field-effect transistors.^57^ This study demonstrated the practical utility of **1-OTf** as a contact-engineering reagent, addressing one of the major bottlenecks in 2D p-type device fabrication. Taken together, these two studies exemplify the continuum from fundamental chemical functionalization to device-level implementation, highlighting how **1-OTf** bridges molecular reactivity with electronic applications in advanced materials (Scheme 15).

**Scheme 15:**
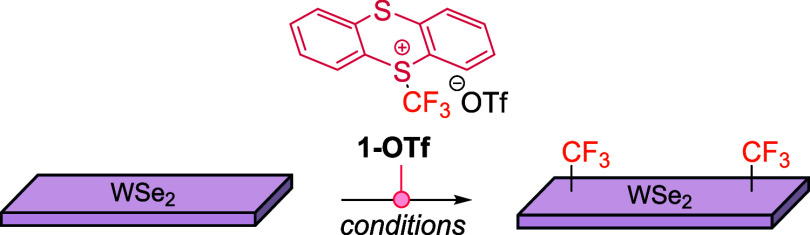
Materials-Oriented Applications of **1-OTf**

## 5 Conclusions

The development of trifluoromethyl thianthrenium salts represents a landmark in the evolution of trifluoromethylating reagents. Its concise one-step synthesis from abundant feedstocks and its exceptional bench stability have provided access to the CF_3_ fragment, previously constrained by multistep or cost-intensive reagents. Although **1-OTf** provides reliable bench stability and broad functional-group compatibility, it is inherently less atom-economical than classical sources such as trifluoroacetic acid, given the larger thianthrene scaffold. Importantly, many reported protocols mitigate this penalty by enabling efficient recovery of the thianthrene byproduct during purification, allowing its recycling in subsequent reagent preparation.

Mechanistic insights reveal that the unique electronic structure of thianthrenium salts underpins their rich reactivity, supporting diverse modes of radical generation through photolysis, electron transfer, and even magnetic induction. Although **1-OTf** provides a uniquely practical platform for CF_3_ transfer, a balanced view of these activation manifolds remains essential. Photochemical homolysis is rapid and operationally simple, whereas EDA processes enable mild, catalyst-free activation but depend on well-matched donor–acceptor pairs. Photoredox, metallaphotoredox, and electrochemical methods offer broad tunability, and magnetoredox activation, while appealing, still requires specialized equipment and remains mechanistically evolving.

From a synthetic perspective, **1-OTf** has proven more than a source of CF_3_^•^: it is a gateway reagent that enables the construction of novel building blocks, including BCP–CF_3_ motifs and trifluoromethoxylation reagents, with direct impact on drug discovery and molecular design. Its compatibility with photoredox, metallaphotoredox, and EDA-based activation further illustrates its versatility across catalytic manifolds, and future opportunities lie in expanding its use in nucleophilic trifluoromethylation chemistry. The translation of **1-OTf** into the materials sciences, where it has been used for the functionalization and defect passivation of TMDs, illustrates its dual nature as both a synthetic and materials reagent as its potential for further functionalization of 2D-semiconductors. Looking forward, we anticipate that the continued exploration of trifluoromethyl thianthrenium salts will not only expand the synthetic landscape of fluorinated motifs but also inspire cross-disciplinary applications at the frontier of catalysis, sustainable synthesis, and electronic materials.

## Data Availability

The data underlying this study are available in the published article.
